# Mammography Rates for Breast Cancer Screening: A Comparison of First Nations Women and All Other Women Living in Manitoba, Canada, 1999–2008

**DOI:** 10.5888/pcd12.140571

**Published:** 2015-05-28

**Authors:** Alain A. Demers, Kathleen M. Decker, Erich V. Kliewer, Grace Musto, Emma Shu, Natalie Biswanger, Katherine Fradette, Brenda Elias, Jane Griffith, Donna Turner

**Affiliations:** Author Affiliations: Alain A. Demers, Jane Griffith, Epidemiology and Cancer Registry, CancerCare Manitoba, Winnipeg, Manitoba, and Department of Community Health Sciences, University of Manitoba, Winnipeg, Manitoba; Kathleen M. Decker, Department of Community Health Sciences, University of Manitoba, Winnipeg, Manitoba, and Screening Programs, CancerCare Manitoba, Winnipeg, Manitoba; Erich V. Kliewer, Epidemiology and Cancer Registry, CancerCare Manitoba, Winnipeg, Manitoba, Department of Community Health Sciences, University of Manitoba, Winnipeg, Manitoba, and Cancer Control Research, British Columbia Cancer Agency, Vancouver, British Columbia; Grace Musto, Emma Shu, Katherine Fradette, Epidemiology and Cancer Registry, CancerCare Manitoba, Winnipeg, Manitoba; Natalie Biswanger, Screening Programs, CancerCare Manitoba, Winnipeg, Manitoba; Brenda Elias, Department of Community Health Sciences, University of Manitoba, Winnipeg, Manitoba.

## Abstract

**Introduction:**

First Nations (FN) women historically have low rates of preventive care, including breast cancer screening. We describe the frequency of breast cancer screening among FN women living in Manitoba and all other Manitoba (AOM) women after the introduction of a provincial, organized breast screening program and explore how age, area of residence, and time period influenced breast cancer screening participation.

**Methods:**

The federal Indian Registry was linked to 2 population-based, provincial data sources. A negative binomial model was used to compare breast cancer screening for FN women with screening for AOM women.

**Results:**

From 1999 through 2008, 37% of FN and 59% of AOM women had a mammogram in the previous 2 years. Regardless of area of residence, FN women were less likely to have had a mammogram than AOM women (relative rate [RR] = 0.69 in the north, RR = 0.55 in the rural south, and RR = 0.53 in urban areas).

**Conclusions:**

FN women living in Manitoba had lower mammography rates than AOM women. To ensure equity for all Manitoba women, strategies that encourage FN women to participate in breast cancer screening should be promoted.

## Introduction

First Nations (FN) are the largest indigenous group in Canada, representing 45% of the indigenous and 2% of the Canadian populations (1). In 2011, 114,225 FN people were living in Manitoba (16.6% of the provincial population); of these, 105,815 had registered Indian status (2), which refers to individuals who, under the federal Indian Act, are entitled to Treaty rights (3) (also referred to as “status Indians”). FN groups indigenous to Manitoba include the Ojibway, Cree, Ojibway–Cree, Dakota, and Dene. FN people reside in urban and rural areas including 63 FN communities, some of which are remote and isolated.

Breast cancer deaths peaked in 1986 in Canada, and the age-standardized mortality rate has fallen by 2.4% per year since 2000 (4). This trend is likely due to a combination of factors, including increased mammography screening (5) and the use of more effective therapies (6,7).

In Manitoba, the incidence of breast cancer among FN women is lower than the incidence among all other Manitoba (AOM) women. From 1994 through 1998, breast cancer incidence was 88 per 100,000 for FN women compared with 131 per 100,000 for AOM women. Breast cancer incidence rates have slightly increased since the mid-1990s; by 2004–2008, it was 100 per 100,000 for FN women and 135 per 100,000 for AOM women (8). Breast cancer mortality rates are also lower for FN women than for AOM women and have slightly decreased since the mid-1990s (1994–1998: 29 per 100,000 for FN women and 35 per 100,000 for AOM women; 2004–2008: 23 per 100,000 for FN women and 30 per 100,000 for AOM women).

Similar to visible minority or new immigrant women, FN women historically have lower rates of preventive care, including breast cancer screening. In the 1990s, screening mammography rates among FN women aged 50 to 69 years were approximately half those of AOM women (26% vs 56%) (9). To improve breast cancer screening rates and decrease the mortality from breast cancer, an organized, province-wide, breast-cancer screening program was initiated in 1995–1996. The program, BreastCheck, invites women aged 50 to 69 years for a bilateral, 2-view mammogram every 2 years. Women with an abnormal mammography result are referred for a diagnostic mammogram, ultrasound, or core biopsy. The program provides screening through 4 fixed sites and, as of 1999–2000, 2 mobile mammography units that visit approximately 55 rural and northern communities each year, including most FN communities. Additional strategies, such as charter flights or group trips to the nearest mobile screening site, are used to further increase access to screening for women living in remote northern communities. 

The objectives of this investigation were to describe the frequency of breast cancer screening from 1999 through 2008 among FN women living in Manitoba compared with that of AOM women after the introduction of BreastCheck and to explore how age, area of residence, and time period influenced breast cancer screening participation.

## Methods

Three population-based data sources were used for this study. The federal Indian Registry is the official record of registered Indians in Canada and contains a list of status Indians (presumed alive or dead) as defined by the federal Indian Act (3). One study investigator (B.E.) led the negotiation with Aboriginal Affairs and Northern Development Canada (the federal data steward) and obtained authorization to link data from the federal Indian Registry to those of the Manitoba Health Population Registry (MHPR). The MHPR is a record of all Manitoba residents who are eligible for insured health services in the province (approximately 99% of the population). A multistep data linkage process identified registered FN individuals in the MHPR file. The Indian Registry file contained 143,274 records with a Manitoba address for the 1999 to 2008 period; 133,882 (93.4%) were successfully linked to the MHPR. AOM women included all women who did not link to the Indian Registry.

A deidentified file using a scrambled identifier and containing information on both FN and AOM women was linked to the Medical Claims database. The provincial Medical Claims database is generated by claims filed by physicians for payment of services and includes a billing tariff code, service date, an *International Classification of Diseases, 9th Revision, Clinical Modification* (ICD-9-CM) diagnosis code, and provider identification. By linking the FN file to the Medical Claims database, women who had a mammogram through the screening program or a bilateral mammogram through a diagnostic facility were identified. A woman was considered screened if she had at least 1 medical claim for a screening program mammogram or a bilateral mammogram in a 2-year period. If a woman had more than 1 screening or bilateral mammogram in a 2-year period, the most recent mammogram was selected. Mammograms performed through the screening program have their own tariff code, but approximately 11% of women have a bilateral mammogram outside the screening program (10). Although we do not know for certain if these bilateral mammograms were for screening or were diagnostic, we included them because they are included when determining the annual percentage of the population that was screened.

The linkage process between the Indian Registry and the MHPR used deterministic and semideterministic criteria. All linkages were matched on 3 or more fields, which were expected to provide a significant degree of differentiation between records (given name, surname, date of birth, date of death, or treaty number).

Frequencies were used to describe the characteristics of the women in the study. A negative binomial model with an exchangeable correlation structure was used to compare the probability of FN women with the probability for AOM women being screened for breast cancer using mammography. Covariates included in the analysis were age in years (50–54, 55–59, 60–64, and 65–69), period (1999–2000, 2001–2002, 2003–2004, 2005–2006, and 2007–2008), and area of residence (north, rural south, urban). Area of residence was determined using each woman’s 6-digit postal code and the Regional Health Authority in which she lived. Younger women were not included because the Canadian breast screening recommendations are for women aged 50 or older (11). All analyses were conducted in SAS version 9.2 (SAS Institute Inc).

Ethical approvals were received from the University of Manitoba Health Research Ethics Board, the Manitoba Health’s Health Information Privacy Committee, CancerCare Manitoba’s Research Impact Committee, and the Assembly of Manitoba Chiefs Health Information and Research Governance Committee.

## Results

Overall, the distribution of demographic characteristics in 2007–2008 did not change substantially from those in 1999–2000 (Table 1). In 1999–2000, 37.0% of FN and 2.6% of AOM women lived in the north. Fewer FN women lived in the north in 2007–2008 (34.7%) than in 1999–2000, but the percentage of AOM women who lived in this region in 2007–2008 did not change. In both periods, a greater percentage of FN than AOM women were aged 50 to 54, and, on average, FN women were 1 year younger than AOM women (57 vs 58 years).

**Table 1:** Characteristics of First Nations Women and all Other Manitoba Women Aged 50 to 69 Years Living in Manitoba in 1999–2000 and in 2007–2008

Characteristic	1999–2000, No. (%)		2007–2008, No. (%)	
	All Other Manitoba (n = 105,604)	First Nations (n = 4,446)	All Other Manitoba (n = 131,632)	First Nations (n = 6,535)
**Region**				
North	2,762 (2.6)	1,644 (37.0)	3,237 (2.5)	2,270 (34.7)
Rural south	34,730 (32.9)	1,568 (35.3)	43,719 (33.2)	2,289 (35.0)
Urban	68,112 (64.5)	1,234 (27.8)	84,676 (64.3)	1,976 (30.2)
**Age, y**				
50–54	35,709 (33.8)	1,646 (37.0)	41,418 (31.5)	2,484 (38.0)
55–59	26,786 (25.4)	1,174 (26.4)	36,518 (27.7)	1,781 (27.3)
60–64	22,219 (21.0)	944 (21.2)	30,659 (23.3)	1,361 (20.8)
65–69	20,890 (19.8)	682 (15.3)	23,037 (17.5)	909 (13.9)

The Figure shows the mammography rates for FN and AOM women from 1999–2000 to 2007–2008. Overall, 37% of FN and 59% of AOM women had a mammogram in the previous 2 years over the study period. Mammography rates increased from 1995–1996 to 1999–2000 at which time the mobile breast screening units were implemented (data not shown). Mammography rates remained level to 2007–2008. In all areas of residence, mammography rates were lower among FN than AOM women. The difference in the rate of mammography between FN and AOM women remained constant from 1999–2000 (relative rate [RR] = 0.61; 95% confidence interval [CI], 0.55–0.69) to 2007–2008 (RR = 0.62; 95% CI, 0.57–0.67). However, in 2007–2008, the gap between the 2 groups was less in the north (RR = 0.74; 95% CI, 0.71–0.78) than in the rural south (RR = 0.60; 95% CI, 0.58–0.62) or in urban areas (RR = 0.53; 95% CI, 0.48–0.59). Similar differences were observed by age (data not shown).

**Figure:**
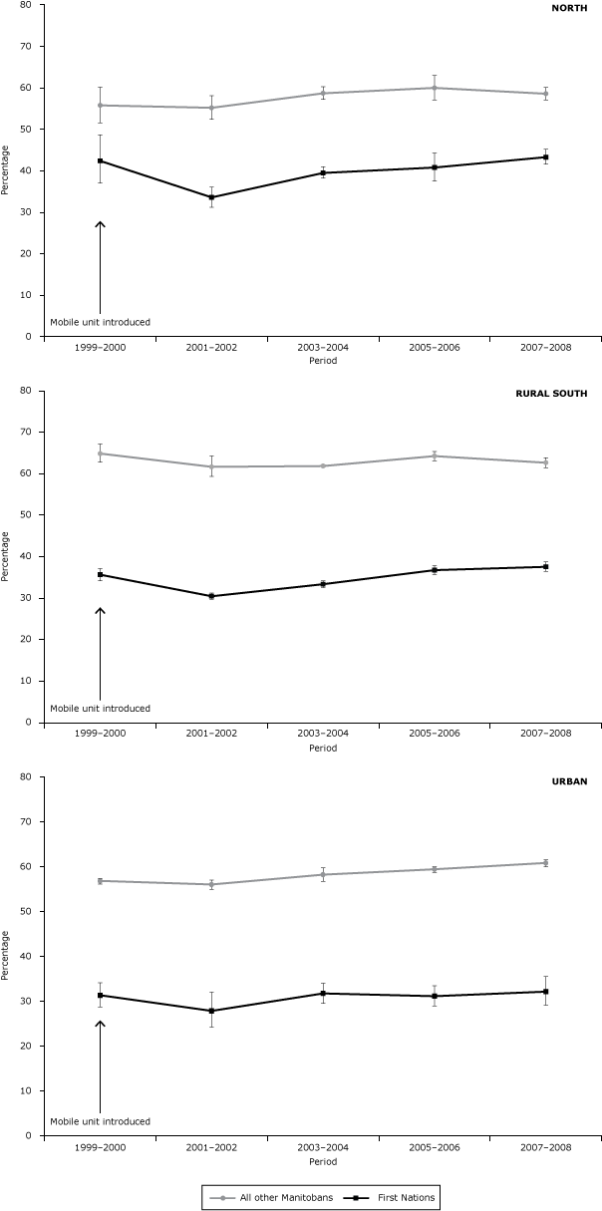
Screening program and bilateral mammography rates among First Nations women and all other Manitoba women, by period and area of residence, 1999–2008. **Table:** 

Area of Residence/Period	First Nations Women	All Other Manitoba Women
	% (95% CI)	
**North**		
1999–2000	42.4 (37.0–48.7)	55.8 (51.6–60.2)
2001–2002	33.6 (31.2–36.1)	55.2 (52.4–58.1)
2003–2004	39.5 (38.2–41.0)	58.7 (57.2–60.3)
2005–2006	40.8 (37.5–44.3)	60.0 (57.1–63.0)
2007–2008	43.3 (41.6–45.2)	58.6 (57.1–60.1)
**Rural south**		
1999–2000	35.6 (33.5–37.9)	64.8 (63.3–66.3)
2001–2002	30.4 (28.1–33.0)	61.6 (60.9–62.4)
2003–2004	33.3 (33.0–33.6)	61.8 (61.0–62.6)
2005–2006	36.7 (35.6–37.8)	64.2 (63.1–65.3)
2007–2008	37.5 (36.3–38.7)	62.6 (61.5–63.8)
**Urban**		
1999–2000	31.3 (28.7–34.1)	56.8 (56.1–57.4)
2001–2002	27.8 (24.2–32.0)	56.0 (54.9–57.0)
2003–2004	31.7 (29.5–34.0)	58.2 (56.7–59.7)
2005–2006	31.1 (28.9–33.4)	59.4 (58.7–60.0)
2007–2008	32.1 (29.1–35.5)	60.8 (60.0–61.5)

Table 2 shows the relative probability of mammography for FN and AOM women by area of residence, age, and period. In the adjusted model, FN women were significantly less likely to have had a mammogram than AOM women (RR = 0.53; 95% CI, 0.51–0.55). In each area of residence, FN women were less likely than AOM women to have had a mammogram in the previous 2 years (RR = 0.69 in the north, RR = 0.55 in the rural south, and RR = 0.53 in urban areas), although the differences were greater for women living in the rural south and urban areas than in the north. Age was not associated with the probability of getting a mammogram. Women were less likely to have had a mammogram in 2001–2002 than in 1999–2000 (RR = 0.83, 95% CI, 0.78–0.89 for FN women; RR = 0.97, 95% CI, 0.95–0.99 for AOM women), although overall, there was little change in mammography rates over time.

**Table 2:** Relative Probability of Having Screening or a Bilateral Mammogram for First Nations Women and all Other Manitoba Women^a^

Characteristic	Crude Model	Adjusted Model
	Relative Rate (95% Confidence Interval)	
**Ethnicity**		
AOM	1 [Reference]	
First Nations	0.59 (0.55–0.64)	0.53 (0.51–0.55)
**Area of residence**		
Urban	1 [Reference]	
North	1.08 (0.84–1.38)	0.99 (0.97–1.02)
Rural south	1.09 (0.82–1.45)	1.08 (1.06–1.10)
**Area of residence, by ethnicity**		
North: AOM	1 [Reference]	
North: First Nations	0.70 (0.65–0.74)	0.69 (0.66–0.74)
Rural south: AOM	1 [Reference]	
Rural south: First Nations	0.55 (0.54–0.57)	0.55 (0.54–0.57)
Urban: AOM	1 [Reference]	
Urban: First Nations	0.53 (0.51–0.55)	0.53 (0.51–0.55)
**Age, y**		
50–54	1 [Reference]	
55–59	0.99 (0.74–1.32)	0.99 (0.97–1.01)
60–64	1.00 (0.75–1.34)	1.00 (0.99–1.02)
65–69	0.96 (0.70–1.31)	0.99 (0.96–1.02)
**Period**		
1999–2000	1 [Reference]	
2001–2002	0.92 (0.88–0.96)	0.95 (0.93–0.97)
2003–2004	0.99 (0.96–1.03)	0.99 (0.96–1.02)
2005–2006	1.02 (0.99–1.06)	1.02 (1.00–1.04)
2007–2008	1.04 (1.00–1.08)	1.02 (0.99–1.06)

Abbreviation: AOM, all other Manitoba.

a Includes women 50 to 69 years of age at time of last screening during the study period.

## Discussion

The rate of mammography for FN women living in Manitoba was lower than the rate for AOM women, regardless of area of residence or time period. These findings are consistent with previous research conducted in Manitoba. From 1997–1999, Martens et al found a mammography rate in the previous 2 years of 26% for FN women 50 to 69 years of age compared with 56% for AOM women (9). This finding was echoed by Elias et al who reported a 27% lower mammography rate for FN women living on reserve in Manitoba during 2002–2003 on the basis of self-reported data from 2 national surveys (1). However, their study also found that the inequity in breast screening for FN women compared with AOM women who live in northern Manitoba decreased over time. This finding may be related to the extensive organized mobile screening program that was introduced throughout Manitoba in 1999. Every 2 years, most communities in rural and northern Manitoba were either visited by the mobile van or travel to a mobile site was facilitated by the screening program (for very remote communities). The mobile screening van also visits neighborhoods with low screening rates.

Beyond Manitoba, little is known about breast cancer screening participation in FN women living in Canada. A study of 133 women conducted in New Brunswick reported a mammography rate in the previous 2 years of 65% (12). Using data from the combined 2007–2011 Canadian Community Health Survey, Withrow et al reported that 59% of FN women living off-reserve had a screening mammogram in the previous 2 years compared with 68% for non-Aboriginal women (13). More information exists on breast cancer screening rates for American Indian and Alaska Native (AI/AN) women, although most of the rates are self-reported. Using the National Health Interview Survey, the Centers for Disease Control and Prevention estimated that 69% of AI/AN women had a unilateral or bilateral mammogram in the previous 2 years, as of 2010 (14). Coughlin et al reported that 65% of AI/AN women aged 50 years or older had a mammogram in the previous 2 years (15). Three groups of researchers reported mammography rates of 68% in the previous 2 years among AI/AN women living in California, based on data from the 2001 California Health Interview Survey (16–18).

Others, however, have reported lower breast cancer screening rates. Giuliano et al interviewed 314 southwest AI women living on reservation (Healthy Hopi Women Survey); only 46% of women stated that they had ever had a mammogram, and 26% of women stated that they had a mammogram in the previous 2 years (19). Giroux et al used the 1995 national audit of medical records from a representative sample of patients with type 2 diabetes at all Indian Health Service (IHS) facilities in the United States to estimate breast cancer screening rates (20). Rates of ever receiving a mammogram varied from 35% to 78% (average 50%) at the 12 IHS facilities. A 3-year screening rate of 38% was reported among AI women aged 45 years or older attending IHS clinics in Montana and Wyoming (21). Using 11 years of data from the Behavioral Risk Factor Surveillance System, Cobb et al found that 67.8% of AI/AN women had a mammogram in the previous 2 years; these data were for women aged 40 years or older (22).

Although the rates of breast screening are higher for AI/AN women than for FN women in this study, most of the US studies reported that these populations were underscreened compared with other US ethnic groups. US authors warned about using national averages to summarize screening prevalence for these populations because of wide variations between regions (23).

Our study has limitations. We were able to include data on registered FN women (93% of all FN women living in Manitoba); data on nonstatus Indians were not included because they are not part of the Indian Registry. Furthermore, FN includes several distinct tribal entities and cultural groups, and our analysis did not distinguish among them. This level of information could be attained through tribal grouping, and future research should consider this possibility if numbers are sufficient and agreements with tribal councils are obtained. Reporting by tribe would greatly increase the ability to plan local collaborations designed to improve the health of these populations. For instance, an analysis by Becker and Foxall of health behavior theories applied to breast screening behavior in AI women found that the most effective interventions were those that were theory-driven, were adaptable, and emphasized a tribal community–researcher partnership (24).

Although our study examined large geographical areas, we did not include factors that could explain the observed trends such as income, level of education, or geographic access to screening. The importance of these factors for breast cancer screening participation has been documented in Manitoba FN communities (1). Furthermore, although higher rates of mammography use were observed among AOM women, this group is heterogeneous for many factors (eg, ethnicity, language, being first generation immigrant), and the rates reported may vary substantially among subpopulations. Individual-level data on ethnicity are not available.

Although our understanding of the health of FN populations living in Canada is improving, much needs to be done. Studies like ours should be repeated across Canada, not only for cancer, but for other health conditions. In terms of cancer screening, barriers that FN women experience and the evaluation of strategies used to try to address these barriers should be investigated. Determining whether the follow-up rates of abnormal results is the same for the 2 populations would be useful. The information should be gathered with respect for the FN principles of ownership, control, access, and possession (25).

There is a global debate over the benefits of breast cancer screening. However, it is still recommended in Canada, and the goal of organized breast-cancer screening is to reduce disease mortality at the population level while minimizing the harms of screening (26). This is possible only if population uptake is adequate and higher participation rates, particularly among unscreened women, are associated with greater population benefits (27). Therefore, high participation is considered among the most important factors in determining the success of a screening program and is a key goal of organized screening (28). To ensure equity for all Manitoba women, strategies that encourage FN women to participate in breast cancer screening should be increased. Information about the benefits and risks of screening as well as access to screening services must be available to all members of the population, including FN women, so they may equitably make an informed decision about whether to participate (29).
